# Supplementary material for: PKN1 promotes synapse maturation by inhibiting mGluR-dependent silencing through neuronal glutamate transporter activation
Source: Commun Biol. 2020 Nov 26;3:710. doi: 10.1038/s42003-020-01435-w (PMC7691520; doi:10.1038/s42003-020-01435-w)
Supplement: Supplementary file 1 — Supplementary Information [file 42003_2020_1435_MOESM1_ESM.pdf]

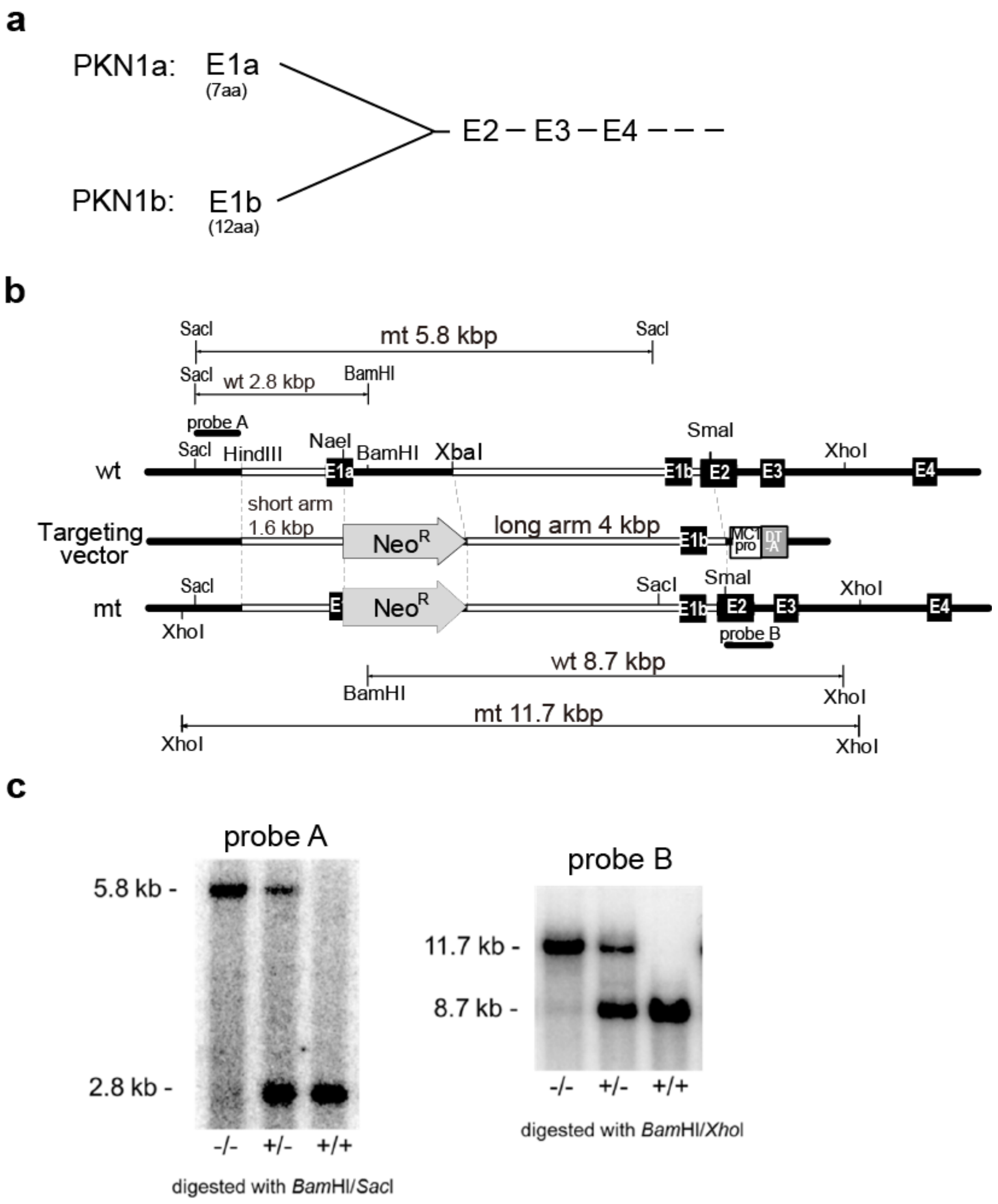

Supplementary Figure 1: Gene targeting of *PKN1a*.

Two PKN1 variant cDNAs have been identified by molecular cloning. These two variants differ in their N-terminal amino acid (aa) sequences and were hypothesized to result from alternative splicing: the classical PKN1 designated as PKN1a was produced from exon 1a encoding the N-terminal 7 aa, and the other PKN1 designated as PKN1b was produced from exon 1b encoding the N-terminal 12 aa, both linking to the common exon 2 and the following exons. (**a**) Schematic exon structure of alternatively spliced PKN1a mRNA. Two

alternative transcripts that seemed to be driven by separate first translating exons (E1a and E1b) and spliced to the common exon 2 are shown. (**b**) Scheme of PKN1 genomic DNA, targeting vector, and disrupted gene. Shown is the targeting vector and a partial map of the PKN1 locus before and after homologous recombination in ES cells. Subsequent breeding of heterozygous mice indicated as PKN1a +/- generates PKN1a KO mice (PKN1a -/-). (**c**) Southern blot analysis. Shown is the result of a representative litter of F2 mice obtained by crossing a pair of PKN1a +/- F1 mice. Genomic DNA was digested with *Bam*HI/*Sac*I and probed with probe A (left) and digested with *Bam*HI/*Xho*I and probed with probe B (right). The positions of probes A and B are illustrated in (**b**).

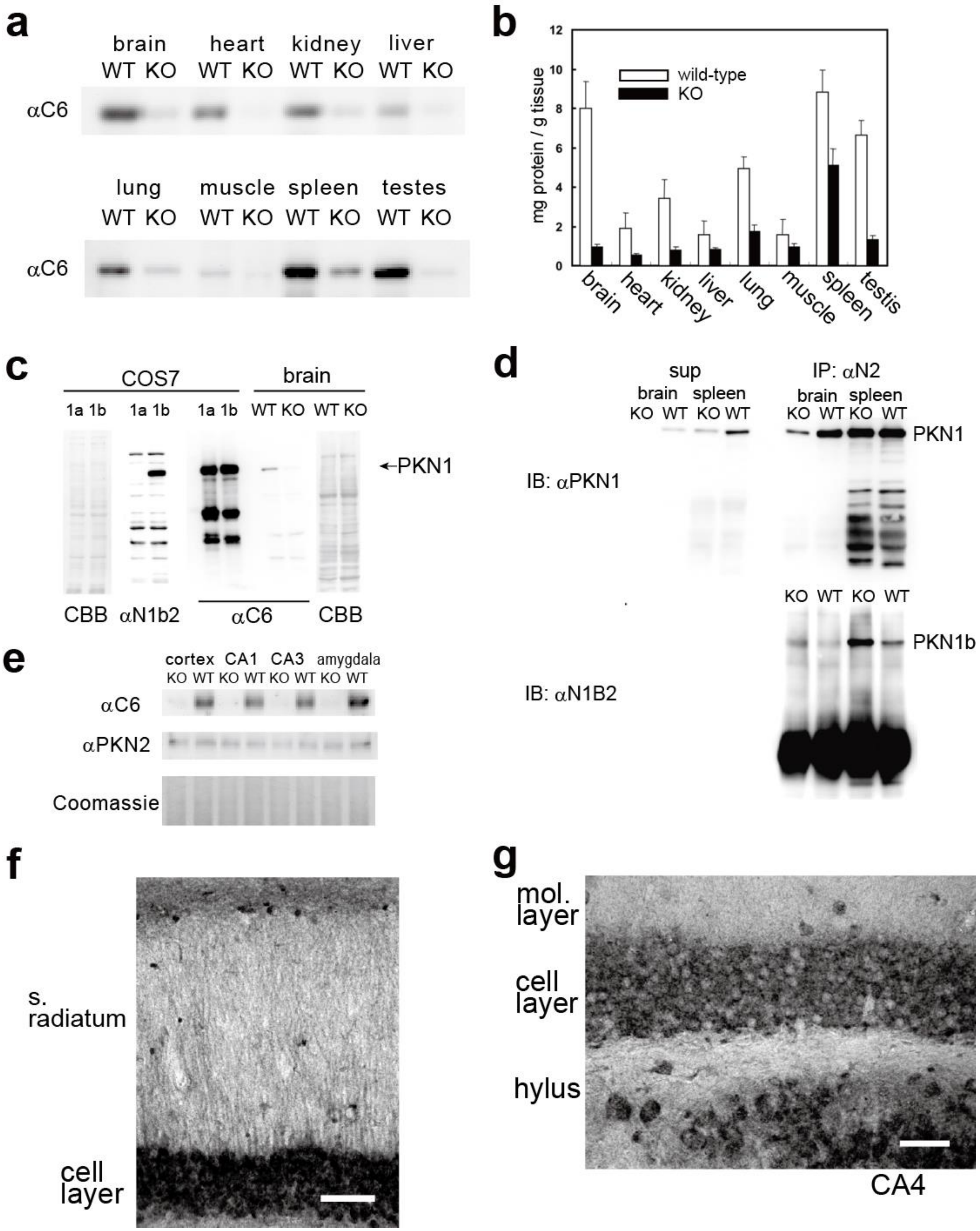

Supplementary Figure 2: Distribution of PKN1 in whole body and brain, and specific deletion of PKN1a, subtype of PKN1, in generated mice.

(**a**) Expression of PKN1 in wild-type and PKN1a KO mice. Whole-cell lysates were prepared from wild-type and PKN1a KO mice, and subjected to immunoblot analysis using the αC6 antibody. WT, wild-type mice; KO, PKN1a KO mice. (**b**) Quantification of PKN1 in various regions of wild-type and PKN1a KO mice. The amount of PKN1 was measured by immunoblot analysis using the αC6 antibody. The data are expressed as the mean ± s.e.m from n = 3 mice per genotype. (**c**) The αN1b2 antibody specifically recognizes PKN1b. Crude lysates were prepared from COS7 cells transfected with PKN1a or PKN1b expression vector. Equal amounts of protein were loaded on all lanes and detected using the αN1b2 antibody or αC6 antibody. (**d**) PKN1b is expressed in PKN1a KO mice. The brain and spleen supernatant from mice (left) and immunoprecipitates from the supernatants prepared using αN2, which is a variant nonselective anti-PKN1 antibody (right) were subjected to immunoblot analysis using a monoclonal antibody against PKN1 (BD Transduction Laboratories) or the αN1b2 antibody. The sensitivity of the αN1b2 antibody is not sufficient to detect endogenous PKN1b in whole-cell lysates. However, PKN1b was detected using the αN1b2 antibody more clearly in αN2 immunoprecipitates from PKN1a KO mice than in those from wild-type mice. (**e**) PKN2 expression in various regions of the mouse brain in wild-type and PKN1a KO mice. Crude homogenates were prepared from various brain regions of mice and subjected to immunoblot analysis using the α ParN2 antibody or αC6 antibody. No compensatory increase in PKN2 expression level was detected in the cortex, the CA1 region of the hippocampus, and the amygdala in PKN1a KO mice. (**f, g**) Immunohistochemical staining of PKN1 in CA1 (f) and the dentate gyrus (g) of the hippocampus in wild-type mice using the αC6 antibody. PKN1 signals were detected in cell layers in the CA1 region and dentate gyrus, and dendrite-like processes in the stratum radiatum of the CA1 region. Scale, 50 μm.

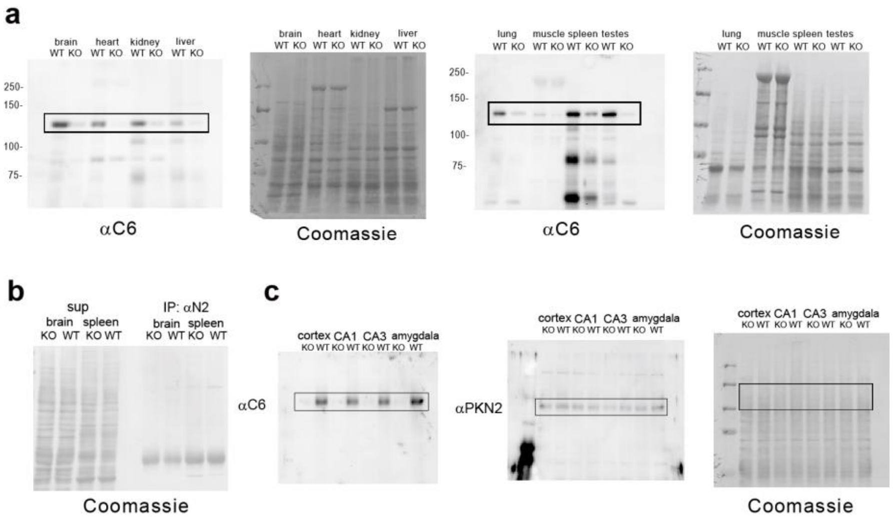

Supplementary Figure 3: Uncropped immunoblot images shown in Supplementary Figure 2.

(**a**) Original images shown in Supplementary Figure 2a and Coomassie staining of the same membranes. (**b**) Coomassie staining of the membrane shown in Supplementary Figure 2d. (**c**) Original images shown in Supplementary Figure 2e and Coomassie staining of the same membrane.

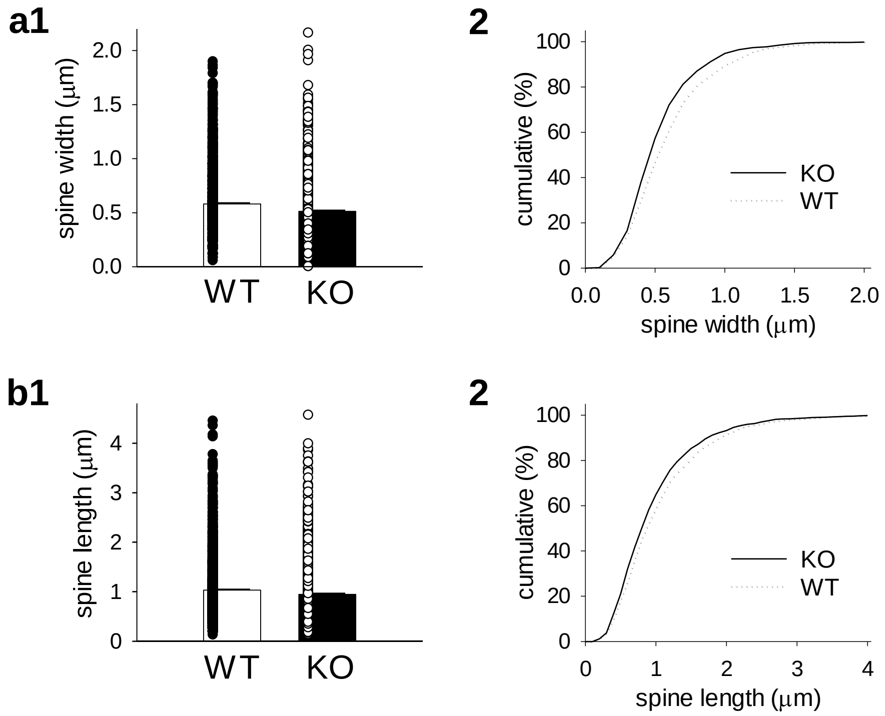

Supplementary Figure 4: Spines in developing hippocampus in PKN1 KO mice are slightly shrunken.

(**a**) Mean (1) and cumulative distributions (2) of spine width on apical dendrites of CA1 pyramidal neurons in wild-type and PKN1a KO mice at P8–15. (**b**) Mean (1) and cumulative distributions (2) of spine length in wild-type and KO mice. Spine width and length were slightly, but significantly, smaller in KO mice than in wild-type mice (width, wild-type, 0.583 ± 0.008 μm, n = 1706 spines in 30 neurons from 3 mice; KO, 0.514 ± 0.008 μm, n = 1262 spines in 32 neurons from 3 mice; $p = 0.0000000019$; length, wild-type, 1.036 ± 0.016 μm; KO, 0.955 ± 0.018 μm; $p = 0.00016$; Mann–Whitney U test).

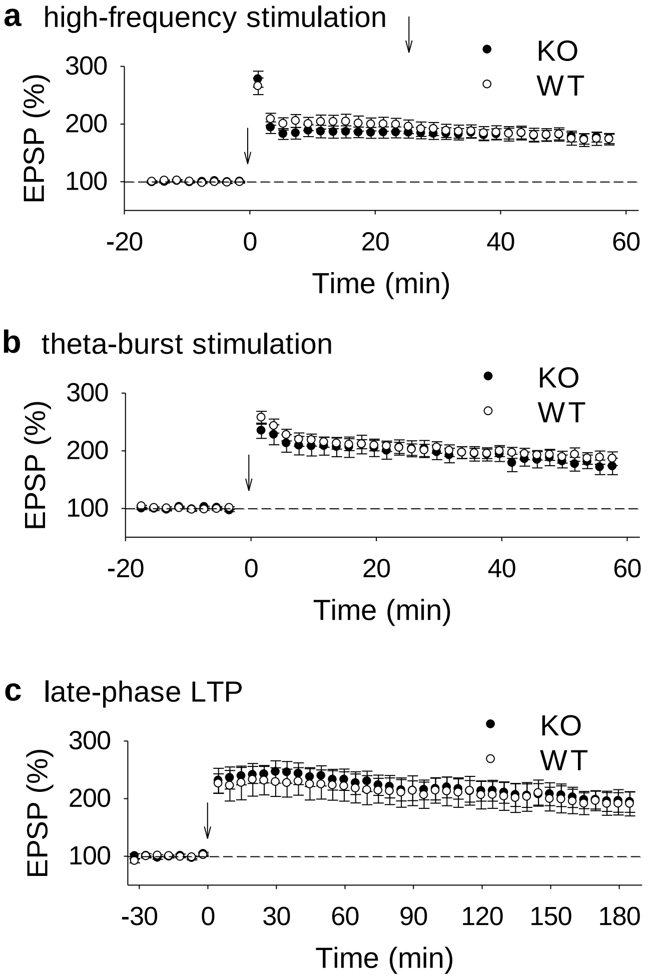

Supplementary Figure 5: Similar LTP is induced in both wild-type and PKN1a KO mice.

(a) Summary of LTP induced by two high-frequency stimulation (HFS, 100 Hz 1 s) in P12-13 wild-type (180.6 ± 10.9% of baseline 50 min after HFS, n = 15 from 3 mice) and PKN1a KO mice (180.6 ± 9.0%, n = 15 from 4 mice). (b) Summary of LTP induced by theta-burst stimulation (TBS, a train of 5 bursts (5 pulses at 100 Hz) at 5 Hz was repeated 4 times every 20 seconds) in P10–18 wild-type (186.0 ± 9.2%, n = 14 from 6 mice) and KO mice (187.4 ± 11.6%, n = 11 from 4 mice). (c) Summary of late-phase LTP induced by 4 100 Hz 1 s stimulation in P10–16 wild-type (185.3 ± 15.8% of baseline 180 min after HFS, n = 9 from 5 mice) and KO mice (185.9 ± 19.9%, n = 9 from 5 mice). Data are shown as mean ± s.e.m.

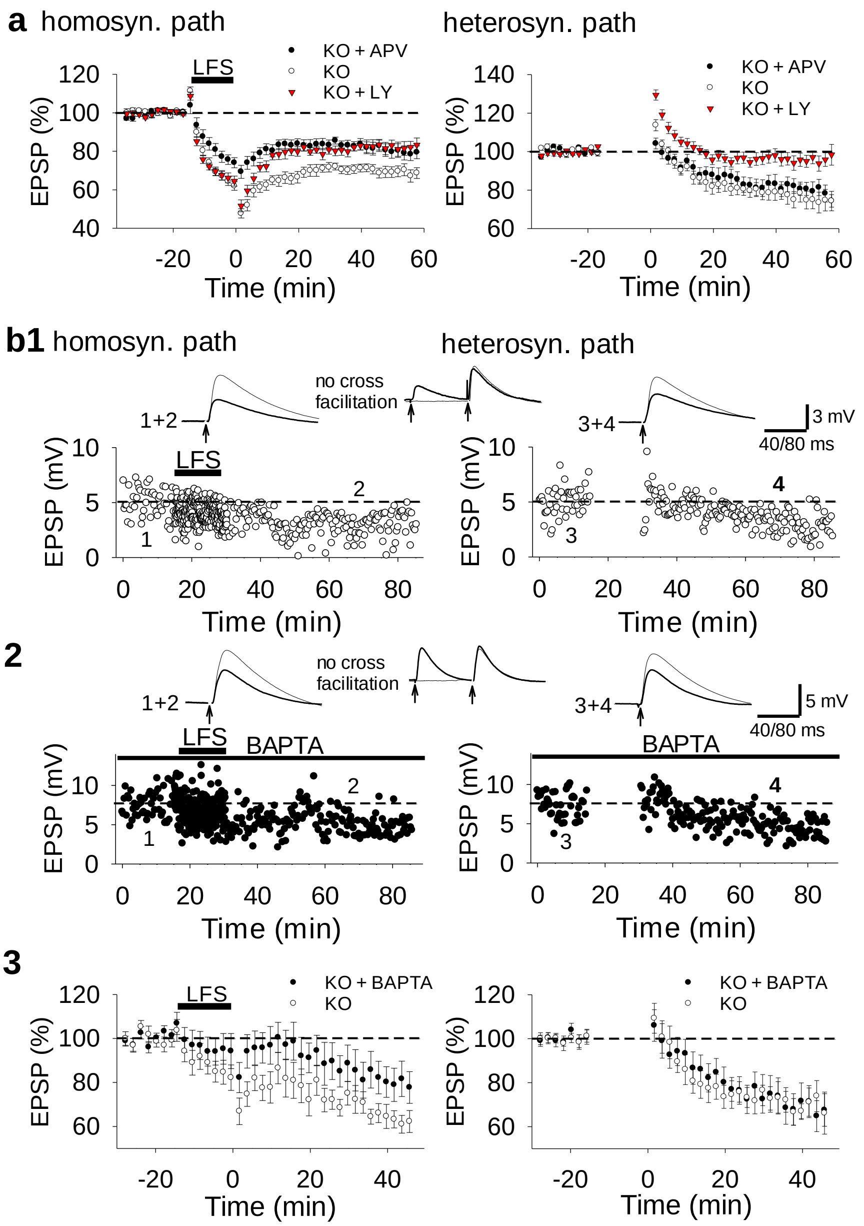

Supplementary Figure 6: Additional LTD abnormally induced in PKN1a KO mice is inhibited by group 1 mGluR antagonist but not by intracellular calcium chelator.

(**a**) Summary of the effects of an NMDAR antagonist D-APV and an mGluR antagonist LY341495 (LY) on homo- (left) and heterosynaptic LTD (right) in P10–14 PKN1a KO mice. D-APV (50 μM) only partially inhibited homosynaptic LTD; unexpectedly, LY341495 (100 μM) also inhibited homosynaptic LTD (no drug, 69.0 ± 2.7%, n = 12 from 11 mice; D-APV, 80.5 ± 3.1%, n = 12 from 7 mice, $F_{(2, 31)} = 6.55$, $p = 0.018$;

LY341495, 82.7 ± 2.7%, n = 10 from 4 mice; *p* = 0.0069; one-way ANOVA with Tukey–Kramer test). D-APV showed no effects; however, LY341495 suppressed heterosynaptic LTD in KO mice (no drug, 76.1 ± 4.5%; APV, 80.2 ± 3.5%; LY, 95.3 ± 3.1%, $F_{(2, 31)}$ = 6.39, *p* = 0.0047; one-way ANOVA with Tukey–Kramer test). (**b**) Examples (1, 2) and summary (3) of the effects of intracellularly loaded BAPTA on homo- and heterosynaptic LTD in P12–16 PKN1a KO mice. BAPTA (10 mM) partially inhibited homosynaptic LTD (no drug, 65.6 ± 3.0% of baseline 40 min after LFS, n = 10 from 8 mice; BAPTA, 84.4 ± 7.1%, n = 16 from 11 mice; $t_{(20)}$ = -2.43, *p* = 0.025; Welch's t test). We consider that calcium-dependent component of homosynaptic LTD in PKN1 KO mice is NMDAR-dependent, because NMDAR-LTD is mediated by calcium. Heterosynaptic LTD was not suppressed by BAPTA (no drug, 70.3 ± 4.6%; BAPTA, 69.8 ± 7.5%), suggesting that mGluR-LTD in the hippocampus in PKN1 KO mice is calcium-independent. All data are shown as mean ± s.e.m.

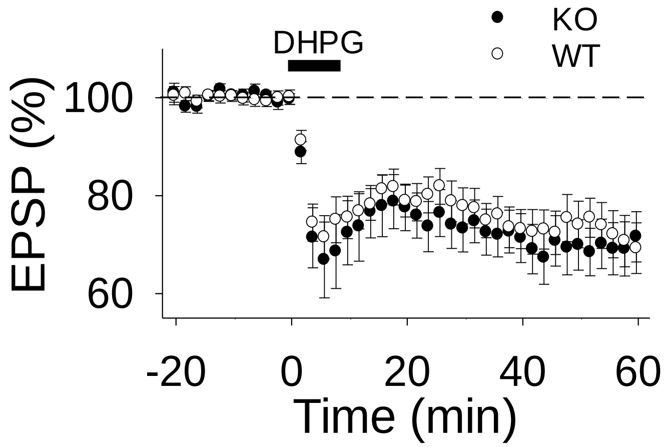

Supplementary Figure 7: Agonist-induced mGluR-LTD is not enhanced in PKN1 KO mice.

Summary of LTD induced by DHPG (50 μM) in wild-type (71.3 ± 5.0%, n = 14 from 3 mice) and PKN1a KO mice at P12–15 (70.6 ± 5.3%, n = 12 from 3 mice). Data are shown as mean ± s.e.m.

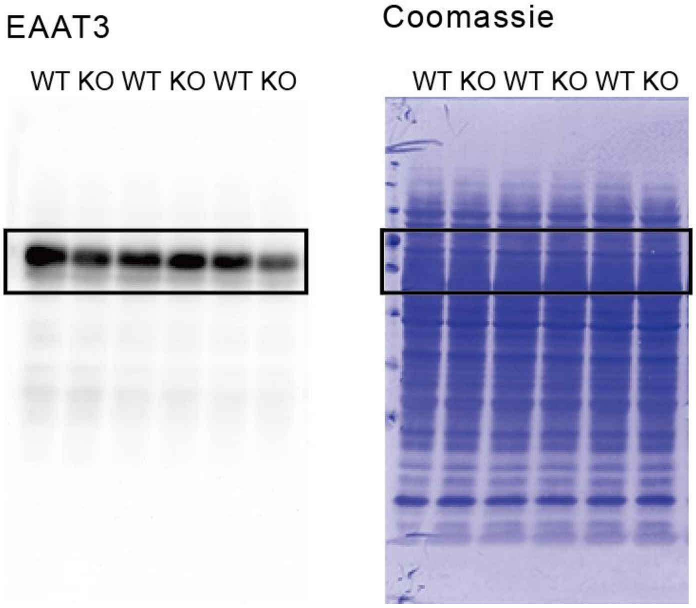

Supplementary Figure 8: Uncropped immunoblot images shown in Fig. 5e.

Original images shown in Fig. 5e and Coomassie staining of the same membranes. A P11–16 wild-type or KO mouse was taken from the home cage at the Animal Facility and immediately anesthetized with isoflurane. Then, the brain was removed and placed in ice-cold oxygenated (95% O_2/5% CO_2) ACSF. The two hippocampi were dissected out, placed in a sample tube, and quick-frozen in liquid nitrogen. Hippocampal lysates were prepared and subjected to immunoblot analysis using an anti-EAAT3 antibody (#12179, Cell Signaling Technology).

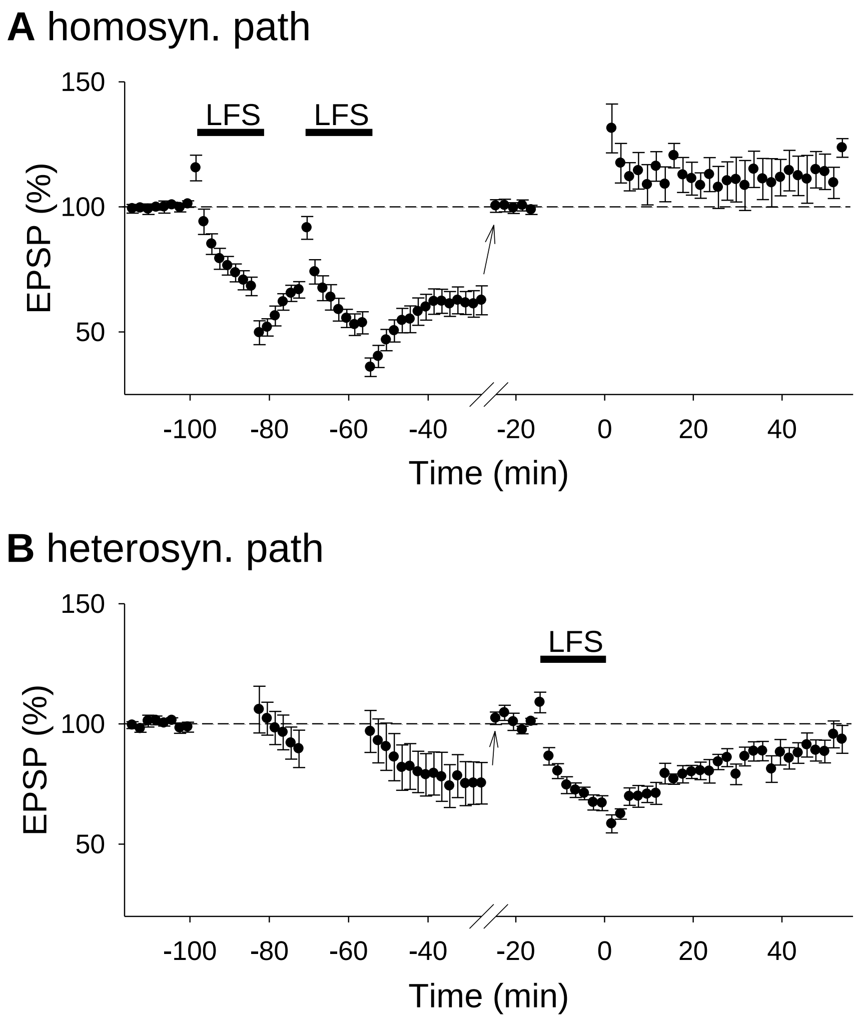

Supplementary Figure 9: Heterosynaptic LTD was occluded by preceding homosynaptic LTD in PKN1 KO mice.

Summary of the effects of preceding homo- and heterosynaptic LTD on hetero- (A) and homosynaptic LTDs in P12–14 KO mice. (B). Heterosynaptic LTD was completely occluded by two LFS (116.0 ± 7.1% of baseline 50 min after third LFS, n = 9 from 5 mice). However, homosynaptic LTD was not completely occluded by heterosynaptic LTD (89.5 ± 4.8%), presumably because NMDAR-dependent LTD was not induced in heterosynaptic pathways by two LFS applied to homosynaptic pathways and there was still room for synaptic depression by NMDA receptor activation in the heterosynaptic pathways when the third LFS was applied. Data are shown as mean ± s.e.m.
