# Supplementary material for: PKN1 promotes synapse maturation by inhibiting mGluR-dependent silencing through neuronal glutamate transporter activation
Source: Commun Biol. 2020 Nov 26;3:710. doi: 10.1038/s42003-020-01435-w (PMC7691520; doi:10.1038/s42003-020-01435-w)
Supplement: Supplementary file 3 — Description of Additional Supplementary Files [file 42003_2020_1435_MOESM3_ESM.pdf]

Description of Additional Supplementary Files

File Name: Supplementary Data 1
Description: Source data behind the graphs in the paper
