# Supplementary material for: PKN1 promotes synapse maturation by inhibiting mGluR-dependent silencing through neuronal glutamate transporter activation
Source: Commun Biol. 2020 Nov 26;3:710. doi: 10.1038/s42003-020-01435-w (PMC7691520; doi:10.1038/s42003-020-01435-w)
Supplement: Supplementary file 5 — Reporting Summary [file 42003_2020_1435_MOESM5_ESM.pdf]

Corresponding author(s): Hiroki Yasuda, Hideyuki Mukai

Last updated by author(s): Jul 30, 2020

# Reporting Summary

Nature Research wishes to improve the reproducibility of the work that we publish. This form provides structure for consistency and transparency in reporting. For further information on Nature Research policies, see our Editorial Policies and the Editorial Policy Checklist.

## Statistics

For all statistical analyses, confirm that the following items are present in the figure legend, table legend, main text, or Methods section.

| n/a | Confirmed | |
|---|---|---|
| ☐ | ☒ | The exact sample size (*n*) for each experimental group/condition, given as a discrete number and unit of measurement |
| ☐ | ☒ | A statement on whether measurements were taken from distinct samples or whether the same sample was measured repeatedly |
| ☐ | ☒ | The statistical test(s) used AND whether they are one- or two-sided<br>*Only common tests should be described solely by name; describe more complex techniques in the Methods section.* |
| ☒ | ☐ | A description of all covariates tested |
| ☐ | ☒ | A description of any assumptions or corrections, such as tests of normality and adjustment for multiple comparisons |
| ☐ | ☒ | A full description of the statistical parameters including central tendency (e.g. means) or other basic estimates (e.g. regression coefficient) AND variation (e.g. standard deviation) or associated estimates of uncertainty (e.g. confidence intervals) |
| ☐ | ☒ | For null hypothesis testing, the test statistic (e.g. *F*, *t*, *r*) with confidence intervals, effect sizes, degrees of freedom and *P* value noted<br>*Give P values as exact values whenever suitable.* |
| ☒ | ☐ | For Bayesian analysis, information on the choice of priors and Markov chain Monte Carlo settings |
| ☒ | ☐ | For hierarchical and complex designs, identification of the appropriate level for tests and full reporting of outcomes |
| ☒ | ☐ | Estimates of effect sizes (e.g. Cohen's *d*, Pearson's *r*), indicating how they were calculated |

*Our web collection on statistics for biologists contains articles on many of the points above.*

## Software and code

Policy information about availability of computer code

| | |
|---|---|
| Data collection | MultiClamp 700A commander (v. 1.3.0.05), MultiClamp 700B commander (v. 2.1.0.16), Igor (v. 7.081), Image J (v. 1.45), Fluochem (v. 3.04A) |
| Data analysis | Igor (v. 7.081), Mini Analysis (v. 6.03), Image J (v. 1.45), Object-Image (v. 2.11), Object J (v. 1.01), Excel, EZR (v. 1.37) |

For manuscripts utilizing custom algorithms or software that are central to the research but not yet described in published literature, software must be made available to editors and reviewers. We strongly encourage code deposition in a community repository (e.g. GitHub). See the Nature Research guidelines for submitting code & software for further information.

## Data

Policy information about availability of data

All manuscripts must include a data availability statement. This statement should provide the following information, where applicable:
- Accession codes, unique identifiers, or web links for publicly available datasets
- A list of figures that have associated raw data
- A description of any restrictions on data availability

The data that support the findings of this study are readily available from the corresponding authors upon reasonable request.

# Field-specific reporting

# Life sciences study design

All studies must disclose on these points even when the disclosure is negative.

| | |
|---|---|
| Sample size | No statistical methods were used to pre-determine sample size. We used sample sizes similar to literature in the field. |
| Data exclusions | For fEPSP recordings, slices with significantly reduced fiber volley amplitudes after LFS were excluded. For whole-cell patch clamp recordings, cells with more than 20% changes in series resistance during recordings were omitted. |
| Replication | The phenotypes were replicated in repeated recordings from different slices from the same and different mice. |
| Randomization | When possible, experimental treatments were randomized. When we examined the effects of various drugs on LTD, for example, we applied these drugs to slices in random order in a day. |
| Blinding | Data collection was not performed blind to the experimental conditions. mEPSC amplitude and frequency, and spine size in golgi-stainded dendrites were analyzed blind to genotype. |

# Reporting for specific materials, systems and methods

We require information from authors about some types of materials, experimental systems and methods used in many studies. Here, indicate whether each material, system or method listed is relevant to your study. If you are not sure if a list item applies to your research, read the appropriate section before selecting a response.

## Materials & experimental systems

| n/a | Involved in the study | |
|---|---|---|
| ☐ | ☒ | Antibodies |
| ☐ | ☒ | Eukaryotic cell lines |
| ☒ | ☐ | Palaeontology and archaeology |
| ☐ | ☒ | Animals and other organisms |
| ☒ | ☐ | Human research participants |
| ☒ | ☐ | Clinical data |
| ☒ | ☐ | Dual use research of concern |

## Methods

| n/a | Involved in the study | |
|---|---|---|
| ☒ | ☐ | ChIP-seq |
| ☒ | ☐ | Flow cytometry |
| ☒ | ☐ | MRI-based neuroimaging |

## Antibodies

| | |
|---|---|
| Antibodies used | abbit anti-N terminal of PKN1a, alphaN2, was synthesized by the authors.<br>Rabbit anti-C terminal of PKN1a, alphaC6, was synthesized by the authors.<br>Rabbit anti-N terminal of PKN1b, alphaN1b2, was synthesized by the authors.<br>Rabbit anti-N terminal of PKN2, alphaParN2, was synthesized by the authors.<br>Anti-PKN1 monoclonal antibody, BD Biosciences Purified Mouse Anti-PRK1 Clone 49/PRK1 Catalog No. 610687 Lot No. 4241750.<br>Rabbit anti-C terminal of EAAT3, Cell Signaling #12179 |
| Validation | AlphaN2 is validated in Mukai H et al., Biochem. Biophys. Res. Commun. 204, 348-356, 1994.<br>AlphaC6 is validated in Mukai H et al., Proc. Natl. Acad. Sci. U S A. 93(19):10195-10199, 1996.<br>Data of AlphaN1b2 is provided in Supplementary Figure 2d in the manuscript.<br>AlphaParN2 is validated in Mukai H et al., Sci. Rep. 6:18979, 2016.<br>Anti-PKN1 monoclonal antibody is validated on https://www.labome.com/product/BD-Biosciences/610687.html.<br>Anti-EAAT3 antibody is validated on https://www.cellsignal.jp/products/primary-antibodies/eaat3-antibody/12179 |

## Eukaryotic cell lines

Policy information about cell lines

| | |
|---|---|
| Cell line source(s) | SH-SY5Y cells |
| Authentication | SH-SY5Y cells were kindly gifted from Prof. Naoaki Saito. |
| Mycoplasma contamination | We did not test for mycoplasma contamination after we got the cells. |
| Commonly misidentified lines<br>(See ICLAC register) | No commonly misidentified cell line was used in the study |

## Animals and other organisms

Policy information about studies involving animals; ARRIVE guidelines recommended for reporting animal research

| | |
|---|---|
| Laboratory animals | Postnatal 7- to 18-day-old (P7–18) male PKN1a KO or their male wild-type littermates were used for this study. They were C57BL/6J (SLC, Japan) genetic background. |
| Wild animals | This study did not use wild animals. |
| Field-collected samples | This study did not use field-collected samples. |
| Ethics oversight | The Ethical Committee for Animal Experiments of Gunma University Graduate School of Medicine, Institutional Animal Care and Use Committee of Kobe University, and the Animal Care and Use Committee of Saga University |

Note that full information on the approval of the study protocol must also be provided in the manuscript.
